# Daylights with high melanopsin stimulation appear reddish in fovea and greenish in periphery

**DOI:** 10.1371/journal.pone.0285053

**Published:** 2023-04-26

**Authors:** Hirokuni Higashi, Katsunori Okajima

**Affiliations:** 1 Graduate School of Environment and Information Sciences, Yokohama National University, Yokohama, Japan; 2 Faculty of Environment and Information Sciences, Yokohama National University, Yokohama, Japan; Federal University of Paraiba, BRAZIL

## Abstract

Melanopsin reportedly contributes to brightness and color appearance in photopic vision in addition to cone photoreceptor cells. However, the relationship between the contribution of melanopsin to color appearance and retinal location is unclear. Herein, we generated metameric daylights (5000 K/6500 K/8000 K) with different melanopsin stimulations while keeping the size and colorimetric values intact and measured the color appearance of the stimuli in the fovea and periphery. The experiment included eight participants with normal color vision. We found that with high melanopsin stimulation, the color appearance of the metameric daylight shifts to reddish at the fovea and greenish in the periphery. These results are the first to show that the color appearance of visual stimuli with high melanopsin stimulation can be completely different in the foveal and peripheral vision even when the spectral power distribution of visual stimuli in both visions is the same. Both colorimetric values and melanopsin stimulation must be considered when designing spectral power distributions for comfortable lighting and safe digital signage in photopic vision.

## Introduction

As the luminous efficiency of color light-emitting diodes (LEDs) continues to improve [1], the design of the spectral power distribution of LED light sources used in lighting and digital signage is expected to become more flexible. Currently, the spectral power distribution of any light source is designed using tristimulus values calculated using color matching functions (CMFs) based on the spectral sensitivity of the three types of cone photoreceptors that are active at the photopic level [2–5]. However, even with constant tristimulus values, the color appearance at the fovea varies with different spectral distributions [6, 7]. Previous CMF studies have investigated the individual differences in the spectral sensitivities of cones [8] and the involvement of photoreceptor cells other than cones [9]. Recent studies have reported that brightness perception in photopic vision is influenced by both melanopsin [10–14] and cones. These studies examined the need to consider the spectral sensitivity function of melanopsin in addition to CMFs when designing spectral power distributions for lighting and signage using LED light sources.

The melanopsin-expressing intrinsically photosensitive retinal ganglion cell (ipRGC), discovered in the early 2000s, was identified as the third photoreceptor [15–17]. IpRGCs receive signals from melanopsin and input from both cones and rods [17–19]. In addition, ipRGCs surround the fovea of the retina with long dendrites [17, 20]. Their density is the highest at approximately 2 mm eccentricity and decreases toward the periphery [21]. Since ipRGCs are expressed in cell bodies, dendrites, and axons [22], it is possible that ipRGCs affect the function of peripheral and foveal vision. Upon first discovery, ipRGCs were initially identified as photoreceptor cells that influence non-image-forming effects such as sleep [23, 24], circadian rhythms [25], and pupillary reflexes [26, 27]. Recent studies have shown that ipRGCs project to the lateral geniculate nucleus [12, 17] and influence the visual pathway for image formation. We focused on melanopsin to clarify whether photoreceptor cells other than cones and rods are involved in visual effects because ipRGCs are affected not only by melanopsin but also by cones and rods. The contribution of melanopsin signals to brightness perception has been reported in human psychophysical studies [10–14]. However, the visual information processing mechanisms of color perception, including melanopsin, have not been elucidated.

Previous research has examined the involvement of photoreceptor cells other than cones in the color appearance in foveal and peripheral vision [28–40]. The color appearance in the periphery is less saturated than that in the fovea, especially for saturations of redness and greenness, which are reduced due to the decrease in cones and the increase in rods with increasing retinal eccentricity [28–36]. Recently, Cao et al. investigated the color appearance in peripheral vision using visual stimuli (30° circular field with the central 10.5° blocked) modulated by melanopsin stimulation while maintaining constant cone and rod stimulation. The authors reported that higher stimulus levels of melanopsin correlated with more greenish colors [37]. Zele et al. and Spitschan et al. reported that orange light with high melanopsin stimulation appeared as orange, bluish-cyan, greenish, and yellowish in the peripheral vision [38, 39]. Photoreceptor cells other than cones reportedly affect the color appearance of the visual stimuli (2° circular field) in foveal vision [40]. Although these studies have suggested that melanopsin and/or rods affect color appearance, the effects in the presentation conditions are unclear because the experiments did not compare color appearance between foveal and peripheral vision.

If the same visual stimulus causes different color appearances between foveal and peripheral vision, color information of the stimulus is not the same depending on the retinal eccentricity. This may cause a risk of errors in judgment based on the color interpretation. If color-mixed LEDs become mainstream, basic data of color appearance dependent on the retinal location are required to design spectral power distributions of light for comfortable lighting and safe digital signage. Barrionuevo et al. have examined the contributions of cones, melanopsin, and rods in color-matching tests at three different field sizes, and their results have shown that the color-matching differences in the extrafoveal and foveal regions may not be explained by rod intrusion, melanopsin may play a role S-cones sensitivity functions in large field [41]. Therefore, our study focused on melanopsin stimulation and presentation conditions of visual stimuli. We generated metameric visual stimuli (1.4° circular field) with different melanopsin stimulations and luminance values while maintaining constant colorimetric values of the reference illuminant and measured the color appearance of the stimuli in the fovea and periphery. The color appearance was evaluated using an elemental color-scaling method to investigate the color shift depending on the experimental conditions.

## Methods

### Apparatus

**Fig 1:**
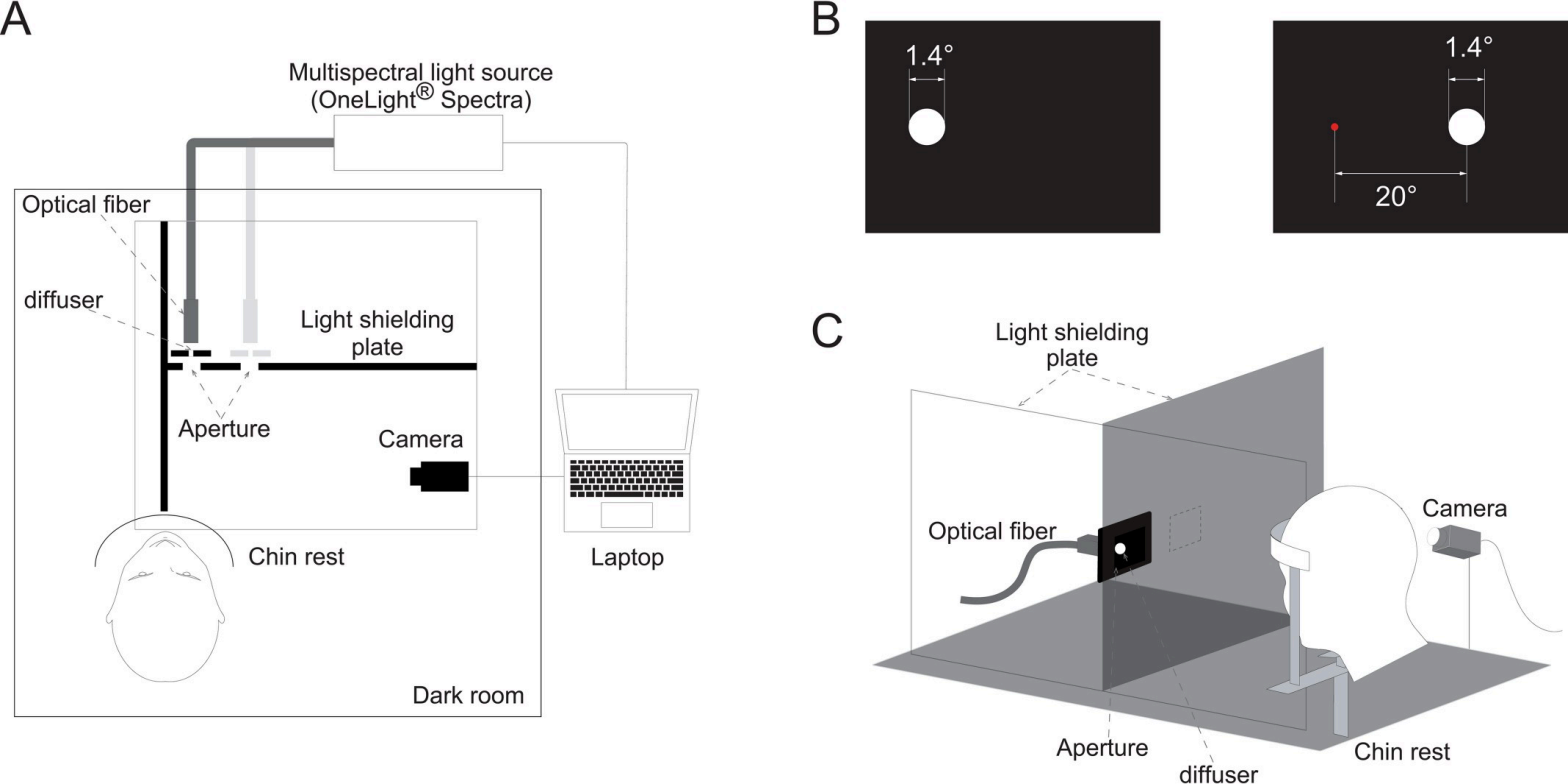
Schematic diagrams of the experimental system. (A) Plan of the experimental space. The visual stimuli were displayed using a multispectral light source. The light originating from the multispectral light source was controlled via a laptop which was located outside the experimental room. Participants were placed in a dark room and asked to observe visual stimuli with the right eye. The head was stabilized with a chin rest. (B) Visual stimuli from the participants’ perspective. The left image shows the view in the foveal vision; the right image shows the view in the peripheral vision. The white circle of each image is the visual stimuli and the red circle is the fixation point. The size of the white circle is 1.4°, and that of the red one is 0.4°. (C) Perth diagram of the experimental space. Visual stimuli were set for the foveal condition. The line of sight of the right eye was checked by the camera. The left eye was occluded by a light-shielding plate to prevent light from entering the eye.

To ensure that no light other than the visual stimuli entered the participants’ fields of view, the experiment was conducted in a dark room with a black-painted experimental setup (Fig 1). Participants’ heads were stabilized with a chin rest and the visual stimuli were generated by a multispectral light source. A light-shielding plate was placed in front of the left eye, and participants observed the visual stimuli with the right eye. The stimuli were presented in either foveal vision (0° in eccentricity) or peripheral vision (20°) by moving the apparatus laterally. An LED with a peak wavelength of 706 nm, size of 0.4°, and luminance of 14 cd/m^2^ was presented as a fixed viewpoint only in the case of the peripheral vision condition. The conditions of the fixed viewpoint were set to minimize the effects of color appearance and intraocular scattering on the visual stimuli presented. We performed a preliminary experiment to confirm that the red light from a fixed viewpoint did not affect the color appearance in the peripheral vision. For proper evaluation of foveal and peripheral vision, eye movement was checked with two USB cameras (HD720p, Logitech Co., Ltd. and ELP-USBFHD06H-MFV, Ailipu Technology Co., Ltd.) during the experiment.

### Participants

Recruited participants included nine healthy volunteers (seven men and two women), aged 24–48 years (average, 30.4; standard deviation, 8.7) with normal color vision and without neurological and ophthalmic disorders. The color vision test was confirmed as normal in all participants using an anomaloscope (OT-II, NEITZ Co., Ltd.) and Farnsworth–Munsell 100 Hue test. One of the nine participants was excluded due to responding “red” in the categorical color naming to the reference illuminant of 5000 K, which is a common white light. The results of the remaining eight participants were analyzed. Among the eight, one participant used a spectacle lens (no color). The study protocol was approved by a formal ethics review board (approval number Hii-2020-14), according to the guidelines of the Yokohama National University Committee on Life Science Research. All participants consented to the experiments in accordance with the Yokohama National University Rules on Life Science Research and provided written consent.

### Visual stimuli

To investigate the effect of melanopsin stimulation on color appearance under photopic conditions, we prepared three conditions in which the tristimulus values were constant and the stimulation level of melanopsin was modulated by applying a silent substitution method [10, 11, 42]. This enabled us to quantitatively investigate the effect of melanopsin stimulation on color appearance. The three conditions for melanopsin stimulation were half-, equal-, and two-fold values of melanopsin stimulation based on the reference illuminant of each correlated color temperature (CCT). The CCT is a measure of the color of a light source. The CCTs of the visual stimuli were set to one orange light stimulus condition (2700 K) and three white light stimulus conditions (5000, 6500, and 8000 K) based on the colors of the visual stimuli evaluated in previous studies. The tristimulus values of each condition were set to be equal to those of the reference illuminants (2700 K for black-body radiation, 5000 K, 6500 K, and 8000 K for [International Commission on Illumination (CIE)] daylight). The tristimulus values were calculated using CIE 2006 2° CMFs. CMFs are sensitivity functions of human vision that are used to calculate the tri-stimulus values. We set up two conditions for the luminance of the visual stimuli: 55 cd/m^2^ and 1,200 cd/m^2^ (rods saturated at ≥5 cd/m^2^) [43].

**Fig 2:**
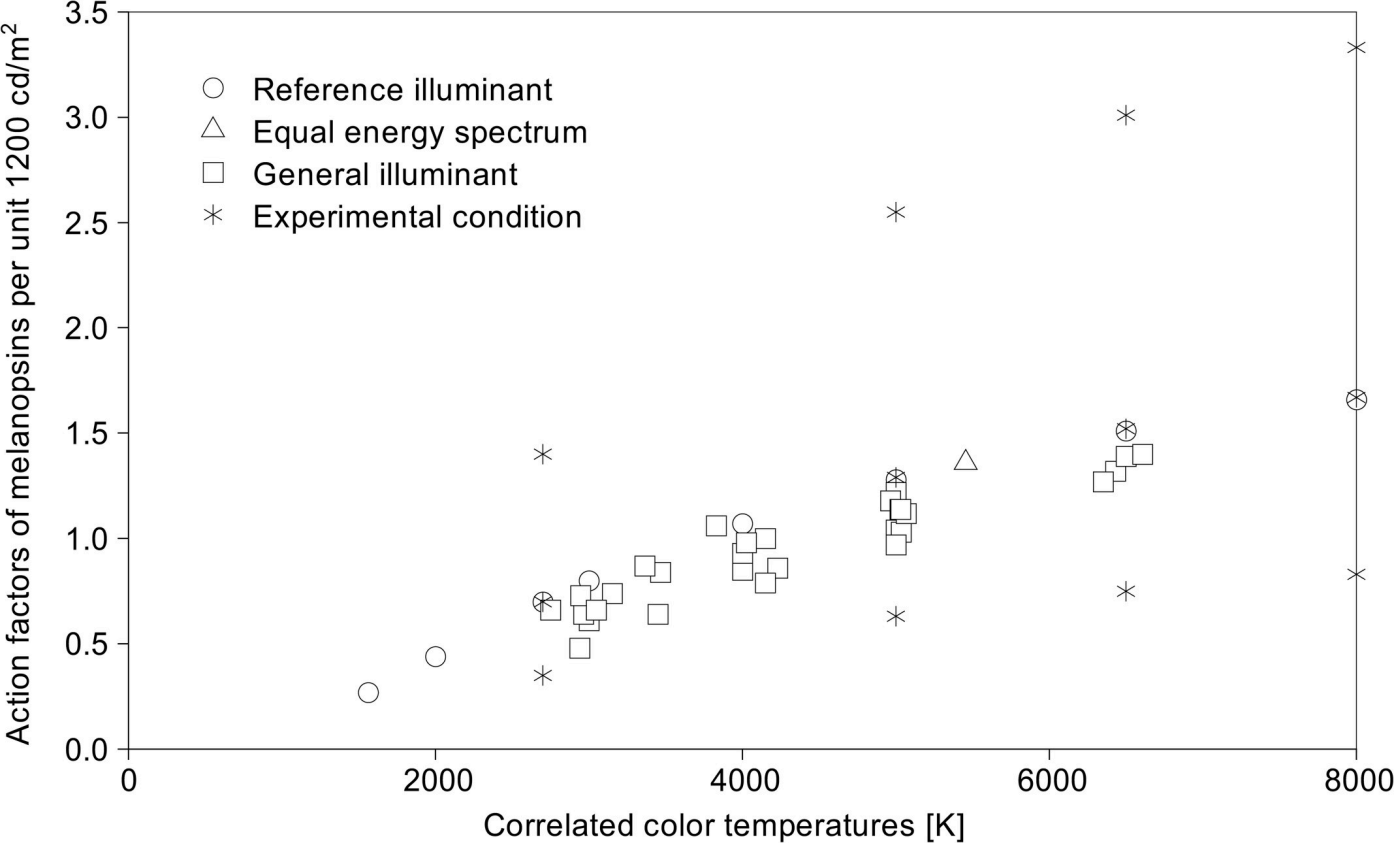
The melanopsin stimulation in the experimental conditions. The relationship between the correlated color temperatures (CCTs) and action factors of melanopsin in several light sources, including the visual stimuli. The reference illuminants are blackbody radiation and CIE daylights. The general illuminants are commonly-used light sources, such as fluorescent lamps and light-emitting diodes (LEDs). The experiment involved four CCT conditions: four in which the stimulation three levels of melanopsin were modulated and the tristimulus values were constant.

The values of the melanopsin stimulation amount used in experimental conditions cover the range of general light sources, such as fluorescent lamps and LED light sources, but the conditions with high stimulation of melanopsin are characterized by the fact that they are very different from general lighting (Fig 2). Our experimental conditions allowed us to confirm the effect of melanopsin stimulation on the color appearance.

The visual stimuli were assessed at two locations: 0° (foveal vision) and 20° (peripheral vision) in eccentricity. The contribution of melanopsin stimulation to color appearance was examined by comparing the results for each stimulus within each of the foveal and peripheral vision conditions. Additionally, by comparing the results of the same stimuli in the foveal and peripheral vision, we were able to quantitatively investigate differences in the color appearance in terms of the retinal position even if the spectral power distribution was the same.

**Fig 3:**
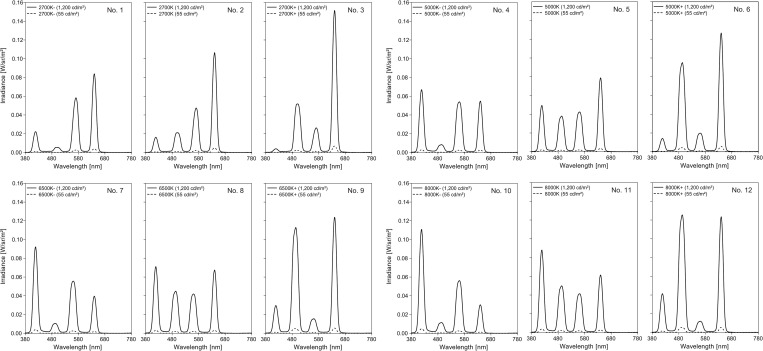
Spectral power distributions of visual stimuli measured using a spectroradiometer. The visual stimulus was generated with a multispectral light source and was presented as a circular transmitted light-emitting uniform surface with a visual angle of 1.4° using a diffuser.

The visual stimulus was generated using a multispectral light source (OneLight Spectra, OneLight Co., Ltd.) with a xenon lamp and was presented circularly with no flicker. For making a transmitted light-emitting uniform surface with a visual angle of 1.4°, we used a diffuser (SW-12; Nikon Co., Ltd.). The multispectral light source output was controlled via a laptop (LATITUDE D630, DELL Inc.). Visual stimuli were measured using a spectroradiometer (SR-3A, Topcon Co., Ltd.). The spectral power distributions of each measured stimulus are shown in Fig 3, and the various values of the visual stimuli are shown in Table 1. The tristimulus values and melanopsin stimulation were calculated from the measured spectral power distributions using the CIE 2006 2° CMF guidelines [4, 5] and the sensitivity function of melanopsin [44] and rods [43] described in the CIE technical report. We modulated melanopsin and rod stimulations simultaneously; designing a spectral power distribution that modulates a large amount of each stimulus independently for similarity of both sensitivities of the melanopsin and rod cells is difficult (S1 Fig). We assumed that the rods were saturated based on the evidence of the CIE publication [43]. We used the values calculated from the CIE 2006 2° CMFs [4, 5]; the recommended values for lighting requirements are frequently calculated using CMFs of 2° [2, 3]. The tristimulus values and chromaticity were calculated using the CIE 2006 10° CMFs [4, 5] and are shown in the S1 Table. The multispectral illumination system was turned on 2 h before the experiment to stabilize the light output. After aging, the spectral power distribution of the visual stimuli was measured. The chromatic value difference between the measured and reference condition chromatic values were confirmed as approximately ±0.002, and recalibration was performed as necessary. The melanopsin stimulation and tristimulus values in the reference conditions were equal values based on the reference illuminant of each CCT.

**Table 1:** Measured values of the visual stimuli.

No.	Condition	*X*_f_	*Y*_f_	*Z*_f_	*Melanopsin*	*Rods*	*x*_f_	*y*_f_
**1**	2700 K-	1,366 (62)	1,212 (55)	367 (16)	0.352 (0.015)	0.562 (0.024)	0.464 (0.467)	0.412 (0.411)
**2**	2700 K	1,361 (62)	1,207 (55)	365 (16)	0.705 (0.032)	0.887 (0.040)	0.464 (0.466)	0.411 (0.412)
**3**	2700 K+	1,357 (62)	1,202 (55)	356 (16)	1.398 (0.063)	1.529 (0.069)	0.465 (0.467)	0.412 (0.413)
**4**	5000 K-	1,164 (53)	1,205 (55)	975 (44)	0.634 (0.028)	0.881 (0.040)	0.348 (0.350)	0.360 (0.361)
**5**	5000 K	1,162 (53)	1,204 (55)	980 (44)	1.293 (0.059)	1.439 (0.066)	0.347 (0.348)	0.360 (0.362)
**6**	5000 K+	1,161 (53)	1,204 (55)	971 (44)	2.561 (0.117)	2.510 (0.114)	0.348 (0.349)	0.361 (0.362)
**7**	6500 K-	1,149 (52)	1,212 (55)	1,312 (59)	0.759 (0.034)	0.995 (0.045)	0.313 (0.314)	0.330 (0.331)
**8**	6500 K	1,142 (52)	1,205 (55)	1,310 (59)	1.521 (0.069)	1.638 (0.075)	0.312 (0.313)	0.330 (0.331)
**9**	6500 K+	1,145 (52)	1,209 (55)	1,305 (59)	3.028 (0.137)	2.913 (0.131)	0.313 (0.314)	0.330 (0.331)
**10**	8000 K-	1,140 (52)	1,204 (55)	1,552 (70)	0.833 (0.038)	1.058 (0.048)	0.293 (0.294)	0.309 (0.310)
**11**	8000 K	1,144 (52)	1,211 (55)	1,564 (70)	1.688 (0.077)	1.784 (0.081)	0.292 (0.293)	0.309 (0.311)
**12**	8000 K+	1,142 (52)	1,217 (55)	1,557 (70)	3.377 (0.151)	3.215 (0.143)	0.292 (0.293)	0.311 (0.310)

Measured values of the visual stimuli. “No.” represents the condition number. The values without parentheses in the table are those under higher-luminance conditions (1,200 cd/m^2^). Values in parentheses are in lower luminance conditions (55 cd/m^2^). The "-", “none,” and "+" mentioned in the conditions denote half-, equal-, and two-fold the values of melanopsin stimulation based on the reference illuminants of each correlated color temperature. The values in the table were calculated using Eqs.1-10 (presented in the supplementary information [S1 File]). The tristimulus values, melanopsin stimulation, rod stimulation, and chromaticity were calculated from the measured spectral power distributions using the International Commission on Illumination (CIE) 2006 2° color matching functions (CMFs) [4, 5], the sensitivity function of melanopsin [44], and that of rods [43].

### Procedure

The color appearance in response to visual stimuli was evaluated using an elemental color scaling method. Elemental color scaling is a subjective evaluation method that allocates 100 points from the observed color to chromatic and achromatic components and allocates the chromatic component to one color component from each of the red-green and yellow-blue channels. For example, the answers would be 40 for the chromatic component, 60 for the achromatic component, 70 for the red component, and 30 for the yellow component.

**Fig 4:**
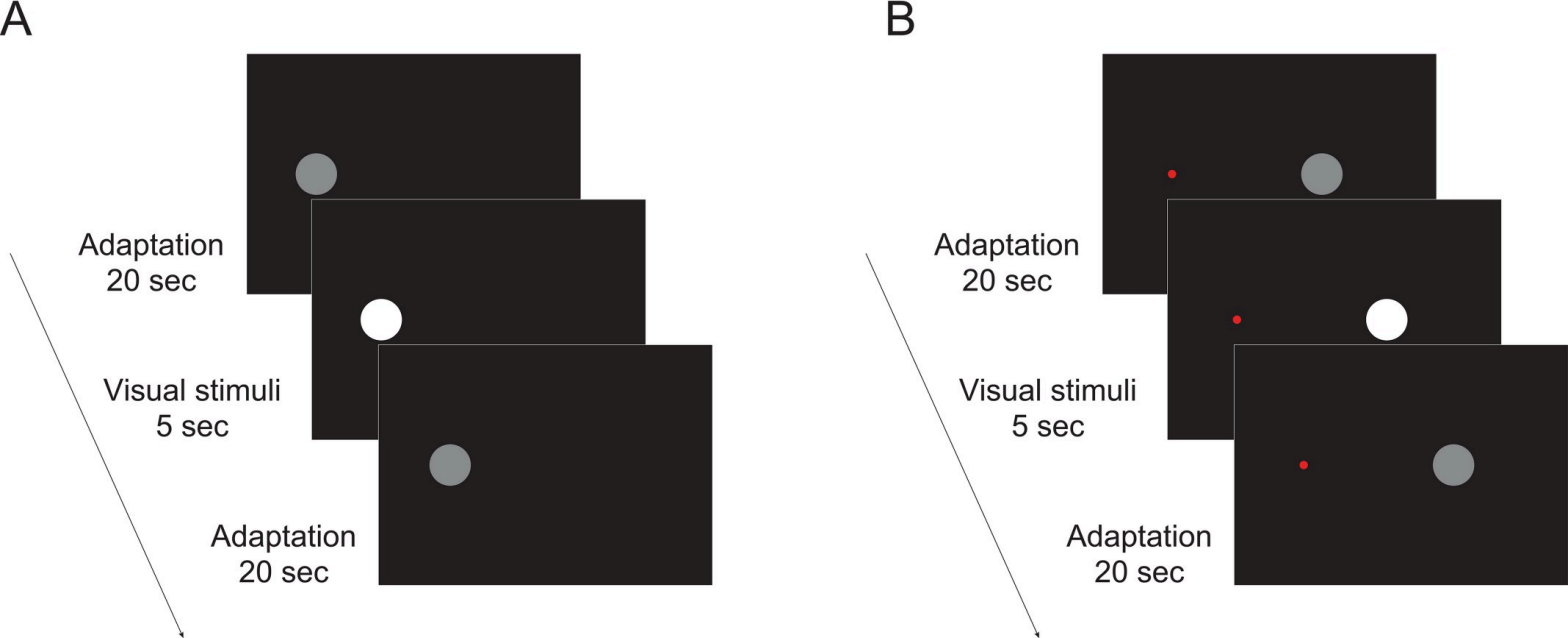
Presentation sequences in the foveal and peripheral vison conditions. (A) Foveal vision. (B) Peripheral vision.

After receiving general instructions and information on experimental precautions and the evaluation methods of color appearance, participants entered the darkroom and performed the evaluation according to the following procedures (Fig 4): (1) participants rested in the dark for 1 min and (2) an adaptive light of approximately 5000 K was presented for 20 s at the same presentation position as the visual stimulus. At this time, participants observed the adaptive light for the foveal condition and the fixation point for the peripheral condition. The luminance of the adaptive light was set to approximately 100 cd/m^2^ when the visual stimulus was 1,200 cd/m^2^ and to approximately 5 cd/m^2^ when the visual stimulus was 55 cd/m^2^. (3) Participants observed the presented visual stimulus for 5 s and (4) adaptive light was presented for 20 s. During this period, participants verbally performed elemental color-scaling for the visual stimuli. (5) Tasks (2)–(4) were repeated five times and (6) tasks (1)–(5) were repeated five times. The visual stimuli were presented in random order to avoid biases. The first two sets were used for foveal vision under higher-luminance conditions, and the following two sets were used for peripheral vision under higher-luminance conditions. The evaluation under lower-luminance conditions was conducted on different days. Since the participants had to perform 288 evaluations (two presentation positions, four CCTs, three melanopsin stimulations, two luminance levels, and six repetitions), the experiment was conducted over two days to minimize the burden on the participants. The experimental time for each day was less than 2 h in the daytime to avoid numerous daily rhythms [45] in the eye as much as possible. The data of the trials in which the gaze moved during the experiment were discarded and re-measured.

### Statistics

The responses of elemental color scaling (red-green and yellow-blue responses) in eight participants were analyzed using the open-source software package JASP version 0.16.4. A two-way Bayesian analysis of variance (ANOVA) was conducted, incorporating the effects of the two luminance conditions (high and low) and three melanopsin stimulation conditions (half-, equal-, and two-fold) on the responses in each CCT and eccentricity condition, with a sample size of eight (average responses of all participants). Bayesian analyses permitted a test of the relative strength of evidence for the null hypothesis (H0: no effect) versus the alternative hypothesis (H1: an effect). The analysis used the inclusion Bayes factor (BF) of the H1 to the H0. The BF quantifies the change from prior inclusion odds to posterior inclusion odds and can be interpreted as evidence in the data for including a predictor. When the BF was >10, it was judged as a significant main effect of the condition or interaction.

## Results

**Fig 5:**
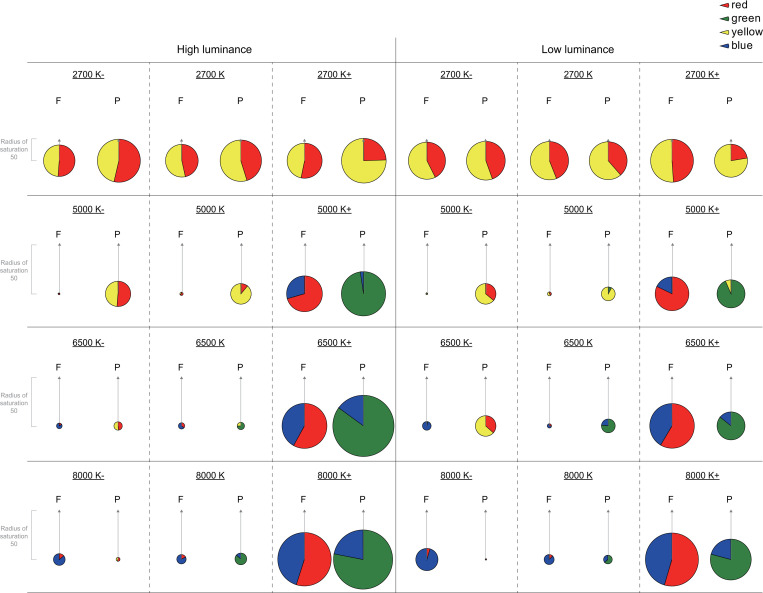
Pie charts were plotted with the results of foveal and peripheral vision in each of the correlated color temperature (CCT) conditions (2700, 5000, 6500, and 8000 K). The left and right graphs show the results of the high (1,200 cd/m^2^) and low (55 cd/m^2^) luminance conditions. The sizes of the pie charts indicate saturation; each portion represents the color components (red, green, yellow, blue) of the elemental color-scaling. The “-,” “none,” and “+” labels correspond to half-, equal-, and two-fold values of melanopsin stimulation based on the reference illuminants of each CCT condition. The “F” and “P” denote foveal and peripheral vision.

Eight participants evaluated the color appearance of visual stimuli with different melanopsin stimulations. Fig 5 shows the appearance shift in the red-green and yellow-blue responses from the foveal vision to the peripheral vision. Both responses indicated that the 2700 K condition vaguely showed hue changes depending on the melanopsin stimulation and eccentricity, whereas the metameric daylight (5000, 6500, and 8000 K) conditions clearly showed hue changes. Specifically, in the red-green response, the visual stimuli of the metameric daylights with the “+” conditions (high stimulation level of melanopsin) tended to be seen as reddish in the foveal vision and as greenish in the peripheral vision. The yellow-blue response showed a gradual increase in blueness with increasing CCT, independent of eccentricity. The “+” conditions tended to shift to more blueness in the foveal and peripheral vision compared to those at the “-”and “none” conditions. No differences in luminance conditions were found in the red-green and yellow-blue responses. The results revealed that even with the same spectral power distributions, the appearance of some colors in the fovea and periphery differed in a contradictory way, red vs. green, depending on the melanopsin stimulation and retinal location.

**Fig 6:**
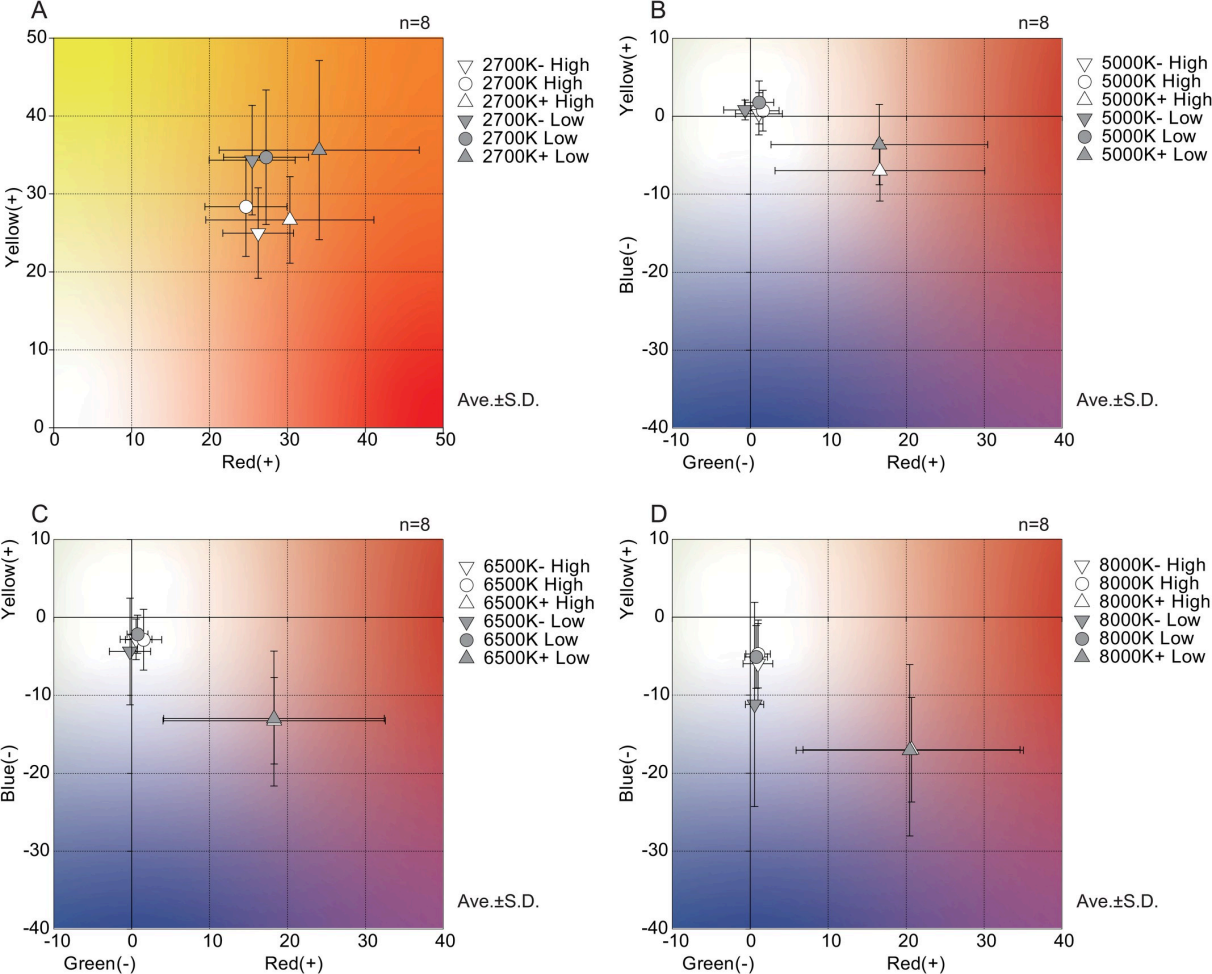
Response results of the elemental color-scaling for eight participants in the foveal vision condition. The correlated color temperatures (CCTs) of the visual stimuli are (A) 2700 K, (B) 5000 K, (C) 6500 K, and (D) 8000 K. The vertical axis represents the +red-green component and the horizontal axis represents the +yellow-blue component. The symbols indicate the conditions of the visual stimuli; the inverse triangle, circle, and standard triangle indicate half-, equal-, and two-fold of the values of melanopsin stimulation based on the reference illuminant of each CCT. “High” and “Low” in the legends denote high (1,200 cd/m^2^) and low (55 cd/m^2^) luminance conditions, respectively.

Fig 6 shows the average results in the foveal vision. The “-,” “none,” and “+” labels correspond to half-, equal-, and two-fold values of melanopsin stimulation based on the reference illuminants of each CCT (2700, 5000, 6500, and 8000 K) and luminance (High: 1,200 cd/m^2^, Low: 55 cd/m^2^). Table 2 shows the analysis of the effects in the two-way Bayesian ANOVA conducted on the results of foveal vision. In the 2700 K condition, there was a significant main effect of luminance on the blue-yellow responses; the other conditions had no effect. Regarding the changes in the main effect, there was a tendency for yellowness to decrease at high luminance. The metameric daylight conditions exhibited a different trend from the 2700 K condition. For the 5000 and 6500 K conditions, there was a significant main effect of melanopsin stimulation on the red-green and blue-yellow responses; the other conditions had no effect. The main effect was a distinctly reddish and slightly bluish color change with increased melanopsin stimulation. At 8000 K, there was a significant main effect of melanopsin stimulation on the red-green responses, whereas the other conditions had no effect. The changes in the main effect were reddish with increased melanopsin stimulation. These results revealed that the color appearance of metameric daylight conditions in foveal vision depended on melanopsin stimulation of the visual stimulus, even under constant chromatic values calculated with the CIE 2006 2° CMFs.

**Table 2:** Bayesian analysis of variance (ANOVA) conducted on the responses of elemental color-scaling (red-green and yellow-blue responses) in foveal vision.

Conditions	2700 K		5000 K		6500 K		8000 K	
	R-G	Y-B	R-G	Y-B	R-G	Y-B	R-G	Y-B
**Lum.**	0.56	205.92	0.32	3.94	0.28	0.25	0.26	0.47
**Mel.**	0.79	0.24	14.41	43.09	24.03	33.36	60.98	3.19
Lum.*Mel.	0.39	0.25	0.33	2.38	0.25	0.28	0.23	0.51

The first column denotes each condition, and the second and subsequent columns show the Bayes factor (BF) values in each correlated color temperature (CCT) and response. The "Lum." and “Mel.” denote the luminance and melanopsin stimulation conditions, respectively. R-G, red-green; Y-B, yellow-blue.

**Fig 7:**
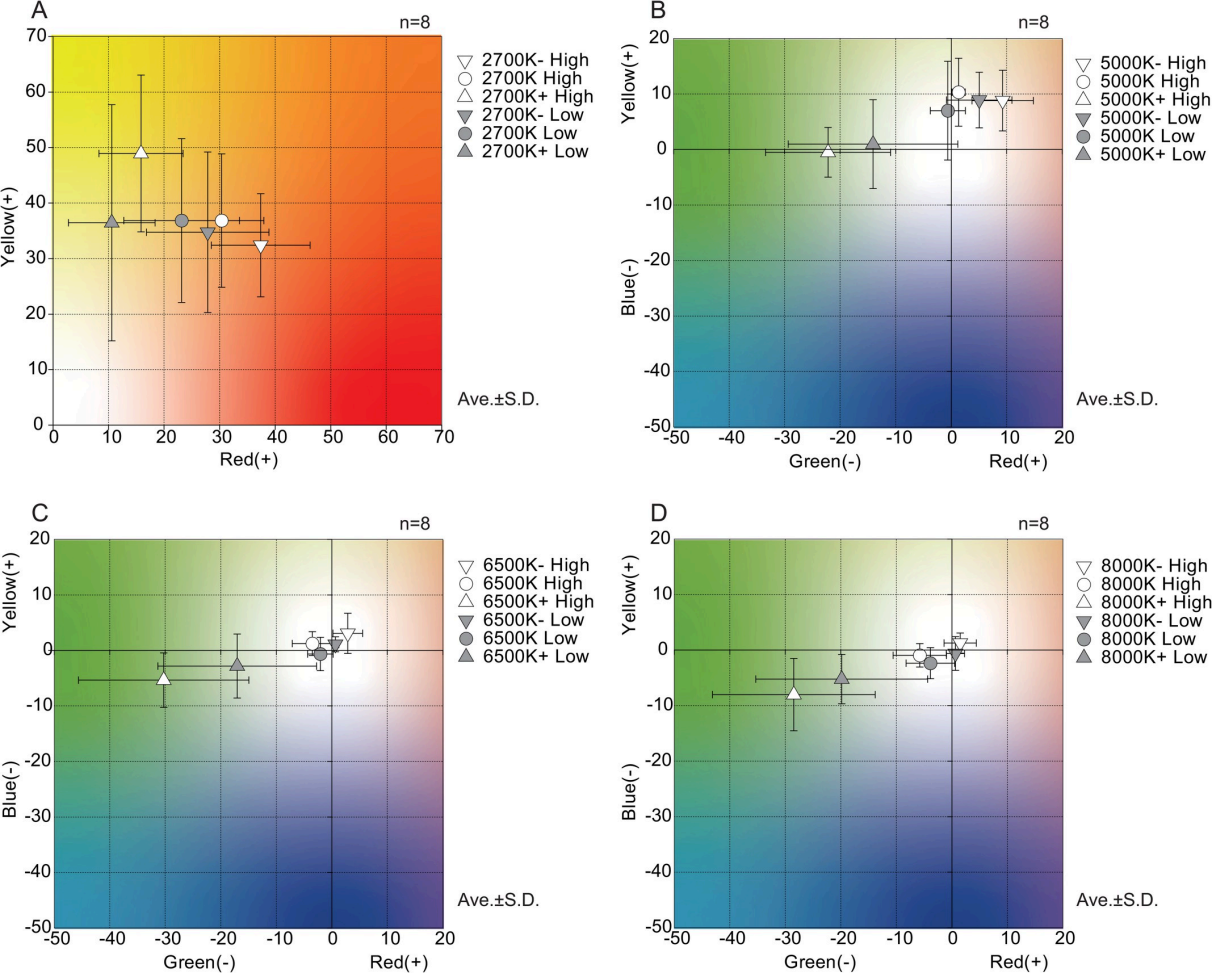
Response results of the elemental color-scaling for eight participants in the peripheral vision condition. The correlated color temperatures (CCTs) of the visual stimuli are (A) 2700 K, (B) 5000 K, (C) 6500 K, and (D) 8000 K. The vertical axis represents the +red-green component and the horizontal axis represents the +yellow-blue component. The symbols indicate the conditions of the visual stimuli; the inverse triangle, circle, and standard triangle indicate half-, equal-, and two-fold of the values of melanopsin stimulation based on the reference illuminant of each CCT. “High” and “Low” in the legends denotes high (1,200 cd/m^2^) and low (55 cd/m^2^) luminance conditions, respectively.

Fig 7 shows the average results for the peripheral vision. Table 3 shows the analysis of the effects in the two-way Bayesian ANOVA conducted on the results of peripheral vision. Similar to the results of foveal vision, there was a difference between the 2700 K condition and metameric daylight conditions. At 2700 K, there was a significant main effect of melanopsin stimulation in red-green, whereas no effect was observed under the other conditions. The reddish color decreased with increasing melanopsin stimulation. While there was a significant effect of melanopsin stimulation in the red-green region at 5000 K and 6500 K, no effect was observed under other conditions. The main effect was a clear shift to a greenish color with higher melanopsin stimulation. At 8000 K, there was a significant main effect of melanopsin stimulation on the red-green and blue-yellow responses, with no effect in other conditions. The main effect was a distinctly greenish and slightly bluish color change with increased melanopsin stimulation. The color appearance of the metameric daylight conditions in the peripheral vision depended on melanopsin stimulation of the visual stimulus, even under constant chromatic values calculated with the CIE 2006 2° CMFs.

**Table 3:** Bayesian analysis of variance (ANOVA) conducted on the responses of elemental color-scaling (red-green and yellow-blue responses) in peripheral vision.

Conditions	2700 K		5000 K		6500 K		8000 K	
	R-G	Y-B	R-G	Y-B	R-G	Y-B	R-G	Y-B
**Lum.**	8.5	0.6	0.5	0.3	1.7	0.5	0.6	0.5
**Mel.**	160.8	0.6	4,645.2	5.7	10,374.2	8.0	2,728.4	16.4
Lum.*Mel.	1.0	0.9	1.5	0.3	3.9	1.4	0.8	1.4

The first column denotes each condition, and the second and subsequent columns show the Bayes factor (BF) values in each correlated color temperature (CCT) and response. The "Lum." and “Mel.” denote the luminance and melanopsin stimulation conditions, respectively. R-G, red-green; Y-B, yellow-blue.

When comparing the red-green and yellow-blue results in each condition for the same participants, seven participants’ responses trended similarly. One participant’s responses were broadly similar to those of the other seven participants’ responses under peripheral conditions but were slightly different under the foveal conditions in that the participant’s responses did not change in the foveal conditions.

## Discussion

To our knowledge, no study has examined the effect of melanopsin stimulation while keeping the colorimetric values and size intact on color appearance by comparing foveal and peripheral vision. For general light sources such as fluorescent lamps and LEDs, the melanopsin stimulation tends to be lower than that of CIE daylights and has an equal energy spectrum, although it varies depending on the CCTs (Fig 2). The conditions in this study were set for higher or lower stimulation doses of melanopsin compared to those of common light sources in each CCT (2700, 5000, 6500, and 8000 K), and the color appearance was evaluated. The results indicated that the 2700 K condition showed a vague change within a similar hue, whereas in the conditions of white lights of 5000, 6500, and 8000 K (metameric daylights), the visual stimuli with a high stimulation level of melanopsin tended to be clearly seen as reddish colors in foveal vision and as greenish colors in peripheral vision despite the luminance level (55 cd/m^2^, 1200 cd/m^2^) and saturated rod response according to the CIE definition. These results indicate that melanopsin [37–39, 46–48] and/or potentially rods [49, 50], contribute to color vision because the distribution of melanopsin/rods on the retina differs from that of cones [17, 51, 52]. However, the most important point we wish to make in this study is that even if the spectral power distributions of the visual stimuli observed in foveal and peripheral vision are the same, the color appearances of the visual stimuli with high melanopsin stimulation in foveal and peripheral vision are sometimes completely different. Our results are the first to report an opposite trend from red/green to green/red in color appearance between focal and peripheral vision when viewed with a single light source. These experimental results suggest that conventional color theory cannot be applied to artificial light sources with high melanopsin stimulation.

The phenomenon that daylight with high melanopsin stimulation causes different visibility in foveal and peripheral vision could be attributed to a variety of variables including melanopsin, rods, cones, and macular pigments. Therefore, we discuss our results in terms of these variables.

We used the silent substitution method [10, 11, 42] to investigate the involvement of melanopsin in color perception. Previous studies have indicated that this method cannot control cone stimulation because of the influence of penumbral cones, which are located in the shadows of blood vessels in the retina [53, 54]. Hence, it is possible that the experimental conditions in our study did not completely control the cone stimulation. However, since it has been reported that the influence of penumbral cones is reflected when the visual stimulus is rapidly flickered (≥4 Hz) [39, 53], it is considered that there is no influence of penumbral cones in our experiment in which the participants were presented with the visual stimulus using steady-light for 10 seconds. Although we did not consider individual differences among the observers that may have induced artifacts, a similar tendency was broadly identified for all participants in the experiments. The results of this study, in which the responses of color appearance in foveal and peripheral vision are the opponent colors, are the data resulting from the presentation of the same visual stimuli with the same spectral power distributions in the foveal and peripheral fields of vision. Thus, problems with penumbral cones and individual differences noted in previous studies were minimized in our study. Thus, we can conclude that melanopsin stimulation affects color perception in foveal and peripheral vision.

Melanopsin is involved in brightness perception [10–14] and color perception [37–39]. Spitschan et al. measured blood-oxygen-level-dependent (BOLD) signals in the primary visual cortex using functional magnetic resonance imaging under visual stimulation that modulated only melanopsin, and showed BOLD signal responses corresponding to the level of melanopsin stimulation [39]. Based on a principal component analysis of the activity of five photoreceptor cells using natural images, Barrionuevo et al. suggested that melanopsin activity contributes to the parvo-cellular pathway (transmitting red-green information) and the konio-cellular pathway (transmitting yellow-blue information) to alter color perception [55]. Cao et al. perimetrically evaluated visual stimuli with silent tristimulus values, silent rod stimulus levels, and modulated melanopsin stimulation and reported that melanopsin stimulus levels significantly affected color perception in red/green information and had no significant effect on color perception in yellow/blue information [37]. If melanopsin stimulation increases the green response, it is possible that it strongly influenced our results of peripheral vision in metameric daylight conditions. Moreover, Spitschan et al. reported that orange light typically appears as yellow-orange in peripheral vision [39], which is similar to our results under the conditions of orange light (2700 K) in the periphery. These studies suggest that melanopsin affects color vision, and no major discrepancy was observed between our results and those of previous studies. In addition, the results obtained under high melanopsin stimulation in the current study support the theory that the melanopsin signals for color perception are strongly involved in the parvo-cellular pathway. One previous study reported that the contribution of melanopsin in peripheral vision may be additive with the signal of M-cones opposing the signal of L-cones in the parvo-cellular pathway (such as [L–{M + I}]) where “I” is for melanopsin activation [37]. Therefore, under the conditions of high melanopsin stimulation in the present experiment, the strong greenish appearance in the peripheral vision can be considered a strong influence of melanopsin.

The visual stimulus of high melanopsin stimulation appears reddish in the foveal vision, which is similar to Maxwell’s spot, an entopic phenomenon that appears as a red spot at the fovea. Maxwell’s spot is related to the difference in tristimulus values between the 10° and 2° CMFs, and a higher cyan primary light is more likely to appear in metameric visual stimuli [56, 57]. Although the color appearance model to explain the phenomenon of Maxwell’s spots is unclear, the absorption of light by the macular pigment [56] and melanopsin stimulation [57] may be involved. The visual stimuli with high melanopsin stimulation used in the current study were spectral power distributions with a high cyan component. Therefore, the strong reddish appearance in the foveal vision can be considered to be related to Maxwell’s spots. As the current colorimetric system considers only cone inputs, our results indicate that the colorimetric system must also consider melanopsin inputs in photopic vision.

Previous studies examining the relationship between melanopsin and color perception have used large visual stimuli of 30° or more [37–39]. This may be because of the low density of ipRGCs [21]. However, since melanopsin is expressed in the cell bodies of ipRGCs and in the dendrites and axons [22], it is expected that the effect of melanopsin on color perception would be obtained even when the size of the visual stimuli is small. The visual stimulus size was set to 1.4° in the current study, and the results corresponded to those of previous peripheral studies under conditions of high melanopsin stimulation. We concluded that the effect of melanopsin stimulation can be seen when the visual size is greater than 1.4°.

We conducted this study under photopic vision conditions, in which the rods are inactive according to the CIE technical report [43]. The spectral sensitivity curves of melanopsin and rods were remarkably similar (S1 Fig), and it was difficult to control each stimulation independently. Since our results were consistent with those of previous studies on melanopsin [37–39], we assume that melanopsin influenced the results. However, the stimulation of melanopsin and that of rods in the experimental conditions were directly proportional to each other, and the involvement of rods cannot be ruled out. Recent studies have stated that rod activities have a profound effect on the L-cone; the contribution of rods can be considered an additive to the L-cone signal [41] and may drive the visual responses of mice to photopic light levels [58]. Previous studies have also reported complex and dynamic interactions between cone and rod signals [59, 60]. These results imply that the rods affect color perception. Further research is necessary to clarify the mechanism of this drastic change in color appearance between the foveal and peripheral vision.

IpRGCs, utilizing melanopsin as a photopigment, respond to light directly through melanopsin and indirectly through mediated synaptic input from cones and rods [17, 61]. It is possible to differentiate the information ipRGCs provide to the brain depending on the amount and ratio of signals from melanopsin, cones, and rods. Therefore, the differences in our results between foveal and peripheral vision and in each condition of the CCT and the melanopsin stimulation might be due to the differences in melanopsin phototransduction and synaptic input from cones and rods in each vision and condition. Although many studies suggest that melanopsin phototransduction within the ipRGCs affects image-forming in vision [10–14, 37–39], further studies regarding the function of melanopsin and that of cones and rods are needed to elucidate the visual mechanisms for ipRGCs.

## Conclusion

We showed that the color appearance in photopic vision is affected by melanopsin stimulation in both peripheral and foveal vision. In addition to CMFs, the sensitivity function of melanopsin may be used to design the spectral power distribution for future lighting and digital signage using LED light sources. Because the enhancement of melanopsin stimulation causes critical differences in color appearance depending on retinal eccentricity, it is necessary to clarify the relationship between spectral power distribution and color appearance while considering tristimulus values and melanopsin stimulation to precisely control color appearance.

## Supporting information

S1 Fig Spectral sensitivities of melanopsin and rods. (DOCX)

S1 Table Measured values of the visual stimuli calculated using the CIE 2006 10° CMFs. (DOCX)

S1 File Equations used in our experiment. (DOCX)

## References

[1] DOE. 2019 Lighting R&D Opportunities. The U.S. Department of Energy. 2019. Available from: https://www.energy.gov/sites/default/files/2020/01/f70/ssl-rd-opportunities2-jan2020.pdf

[2] CIE. Method of measuring and specifying colour rendering properties of light sources. CIE 013.3–1995. International Commission on Illumination; 1995.

[3] CIE. Colorimetry, 4th Edition. CIE 015:2018. International Commission on Illumination; 2018.

[4] CIE. Fundamental chromaticity diagram with physiological axes—Part 1. CIE 170–1:2006. International Commission on Illumination; 2006.

[5] CIE. Fundamental chromaticity diagram with physiological axes—Part 2: Spectral luminous efficiency functions and chromaticity diagrams. CIE 170–2:2015. International Commission on Illumination; 2015.

[6] Sarkar A, Blondé L, Le Callet P, Autrusseau F, Morvan P, Stauder J. A color matching experiment using two displays: design considerations and pilot test results. Proceedings of the Fifth European Conference on Color in Graphics, Imaging and Vision. 2010.

[7] Ohsawa K, Teraji T, König F, Yamaguchi M, Ohyama N. Color matching experiment using 6-primary display. Proceedings of the 3rd International Conference on Multispectral Color Science (MCS’01). 2001; 85–88.

[8] Yamauchi Y, Kawahara T, Nakano Y, Uchikawa K. Metameric matching and its compensation with individual color matching functions. J Vis. 2004;4: 93–93. doi: 10.1167/4.11.93

[9] Danilova MV, Mollon JD. Bongard and Smirnov on the tetrachromacy of extra-foveal vision. Vision Res. 2021; 107952. doi: 10.1016/j.visres.2021.08.007 34625301

[10] Brown TM, Tsujimura S, Allen AE, Wynne J, Bedford R, Vickery G, et al. Melanopsin-Based Brightness Discrimination in Mice and Humans. Curr Biol. 2012;22: 1134–1141. doi: 10.1016/j.cub.2012.04.039 22633808 PMC3509338

[11] Yamakawa M, Tsujimura S, Okajima K. A quantitative analysis of the contribution of melanopsin to brightness perception. Sci Rep. 2019;9: 1–8. doi: 10.1038/s41598-019-44035-3 31110303 PMC6527610

[12] Brown TM, Gias C, Hatori M, Keding SR, Semo M, Coffey PJ, et al. Melanopsin contributions to irradiance coding in the thalamo-cortical visual system. PLoS Biol. 2010;8: e1000558. doi: 10.1371/journal.pbio.1000558 21151887 PMC2998442

[13] Zele A, Adhikari P, Feigl B, Cao D. Cone and melanopsin contributions to human brightness estimation. J Opt Soc Am A. 2018;35: B19–B25. doi: 10.1364/JOSAA.35.000B19 29603934

[14] Zele AJ, Dey A, Adhikari P, Feigl B. Rhodopsin and melanopsin contributions to human brightness estimation. J Opt Soc Am A. 2020;37: A145–A153. doi: 10.1364/JOSAA.379182 32400534

[15] Provencio I, Rodriguez IR, Jiang G, Hayes WP, Moreira EF, Rollag MD. A Novel Human Opsin in the Inner Retina. J Neurosci. 2000;20: 600–605. doi: 10.1523/JNEUROSCI.20-02-00600.2000 10632589 PMC6772411

[16] Lucas RJ, Hattar S, Takao M, Berson DM, Foster RG, Yau K-W. Diminished Pupillary Light Reflex at High Irradiances in Melanopsin-Knockout Mice. Science. 2003;299: 245–247. doi: 10.1126/science.1077293 12522249

[17] Dacey DM, Liao H-W, Peterson BB, Robinson FR, Smith VC, Pokorny J, et al. Melanopsin-expressing ganglion cells in primate retina signal colour and irradiance and project to the LGN. Nature. 2005;433: 749–754. doi: 10.1038/nature03387 15716953

[18] Gooley JJ, Rajaratnam SMW, Brainard GC, Kronauer RE, Czeisler CA, Lockley SW. Spectral Responses of the Human Circadian System Depend on the Irradiance and Duration of Exposure to Light. Sci Transl Med. 2010;2: 31ra33–31ra33. doi: 10.1126/scitranslmed.3000741 20463367 PMC4414925

[19] Lall GS, Revell VL, Momiji H, Al Enezi J, Altimus CM, Güler AD, et al. Distinct Contributions of Rod, Cone, and Melanopsin Photoreceptors to Encoding Irradiance. Neuron. 2010;66: 417–428. doi: 10.1016/j.neuron.2010.04.037 20471354 PMC2875410

[20] Hannibal J, Christiansen AT, Heegaard S, Fahrenkrug J, Kiilgaard JF. Melanopsin expressing human retinal ganglion cells: Subtypes, distribution, and intraretinal connectivity. J. Comp. Neurol. 2017;525: 1934–1961. doi: 10.1002/cne.24181 28160289

[21] Nasir-Ahmad S, Lee SCS, Martin PR, Grünert U. Melanopsin-expressing ganglion cells in human retina: Morphology, distribution, and synaptic connections. J. Comp. Neurol. 2019;527: 312–327. doi: 10.1002/cne.24176 28097654

[22] Hattar S, Liao HW, Takao M, Berson DM, Yau KW. Melanopsin-containing retinal ganglion cells: architecture, projections, and intrinsic photosensitivity. Science. 2002;295: 1065–1070. doi: 10.1126/science.1069609 11834834 PMC2885915

[23] Tsai JW, Hannibal J, Hagiwara G, Colas D, Ruppert E, Ruby NF, et al. Melanopsin as a Sleep Modulator: Circadian Gating of the Direct Effects of Light on Sleep and Altered Sleep Homeostasis in Opn4−/− Mice. PLoS Biol. 2009;7: e1000125. doi: 10.1371/journal.pbio.1000125 19513122 PMC2688840

[24] Cajochen C. Alerting effects of light. Sleep Med. Rev. 2007;11: 453–464. doi: 10.1016/j.smrv.2007.07.009 17936041

[25] Panda S, Provencio I, Tu DC, Pires SS, Rollag MD, Castrucci AM, et al. Melanopsin Is Required for Non-Image-Forming Photic Responses in Blind Mice. Science. 2003;301: 525–527. doi: 10.1126/science.1086179 12829787

[26] Tsujimura S, Ukai K, Ohama D, Nuruki A, Yunokuchi K. Contribution of human melanopsin retinal ganglion cells to steady-state pupil responses. Proc Biol Sci. 2010;277: 2485–2492. doi: 10.1098/rspb.2010.0330 20375057 PMC2894926

[27] Lee S, Hida A, Tsujimura S, Morita T, Mishima K, Higuchi S. Association between melanopsin gene polymorphism (I394T) and pupillary light reflex is dependent on light wavelength. J Physiol Anthropol. 2013;32: 16. doi: 10.1186/1880-6805-32-16 24119231 PMC4015917

[28] Boynton RM, Schafer W, Neun ME. Hue-Wavelength Relation Measured by Color-Naming Method for Three Retinal Locations. Science. 1964;146: 666–668. doi: 10.1126/science.146.3644.666 14191711

[29] Gordon J, Abramov I. Color vision in the peripheral retina. II. Hue and saturation. J Opt Soc Am. 1977;67: 202–207. doi: 10.1364/josa.67.000202 839300

[30] Abramov I, Gordon J, Chan H. Color appearance in the peripheral retina: effects of stimulus size. J Opt Soc Am A. 1991;8: 404–414. doi: 10.1364/josaa.8.000404 2007915

[31] Abramov I, Gordon J, Chan H. Color appearance across the retina: effects of a white surround. J Opt Soc Am A. 1992;9: 195–202. doi: 10.1364/josaa.9.000195 1542060

[32] Ayama M, Sakurai M. Changes in hue and saturation of chromatic lights presented in the peripheral visual field. Color Res Appl. 2003;28: 413–424. doi: 10.1002/col.10194

[33] Mogi S, Sakurai M, Ishikawa T, Ayama M. Color appearance of small stimuli presented in central and near peripheral visual fields. Color Res Appl. 2021;46: 722–739. doi: 10.1002/col.22610

[34] Stabell B, Stabell U. Rod and cone contributions to peripheral colour vision. Vision Res. 1976;16: 1099–1104. doi: 10.1016/0042-6989(76)90249-2 969221

[35] Stabell B, Stabell U. Peripheral Colour Vision: Effects of Rod Intrusion at Different Eccentricities. Vision Res. 1996;36: 3407–3414. doi: 10.1016/0042-6989(96)00079-x 8977008

[36] Stabell U, Stabell B. Rod–cone color mixture: effect of size and exposure time. J Opt Soc Am A. 1999;16: 2638–2642. doi: 10.1364/josaa.16.002638 10546346

[37] Cao D, Chang A, Gai S. Evidence for an impact of melanopsin activation on unique white perception. J Opt Soc Am A Opt Image Sci Vis. 2018;35: B287–B291. doi: 10.1364/JOSAA.35.00B287 29603954 PMC6223255

[38] Zele AJ, Adhikari P, Cao D, Feigl B. Melanopsin driven enhancement of cone-mediated visual processing. Vision Res. 2019;160: 72–81. doi: 10.1016/j.visres.2019.04.009 31078661

[39] Spitschan M, Bock AS, Ryan J, Frazzetta G, Brainard DH, Aguirre GK. The human visual cortex response to melanopsin-directed stimulation is accompanied by a distinct perceptual experience. PNAS. 2017;114: 12291–12296. doi: 10.1073/pnas.1711522114 29087940 PMC5699066

[40] Kagimoto A, Okajima K. Perfect appearance match between self-luminous and surface colors can be performed with isomeric spectra. Sci Rep. 2020;10: 18350. doi: 10.1038/s41598-020-75510-x 33110204 PMC7591860

[41] Barrionuevo PA, Filgueira CP, Cao D. Is melanopsin activation affecting large field color-matching functions? J Opt Soc Am A. 2022;39: 1104–1110. doi: 10.1364/JOSAA.457223 36215541

[42] Estévez O, Spekreijse H. The “silent substitution” method in visual research. Vision Res. 1982;22: 681–691. doi: 10.1016/0042-6989(82)90104-3 7112962

[43] CIE. Recommended system for mesopic photometry based on visual performance. CIE 191:2010. International Commission on Illumination; 2010.

[44] CIE. CIE system for metrology of optical radiation for ipRGC‐Influenced responses to light. CIE S 026/E:2018. International Commission on Illumination; 2018.

[45] Stone RA, Quinn GE, Francis EL, Ying G, Flitcroft DI, Parekh P, et al. Diurnal Axial Length Fluctuations in Human Eyes. Invest. Ophth. Vis. Sci. 2004;45: 63–70. doi: 10.1167/iovs.03-0294 14691155

[46] Horiguchi H, Winawer J, Dougherty RF, Wandell BA. Human trichromacy revisited. PNAS. 2013;110: E260–E269. doi: 10.1073/pnas.1214240110 23256158 PMC3549098

[47] Brown TM, Allen AE, al-Enezi J, Wynne J, Schlangen L, Hommes V, et al. The Melanopic Sensitivity Function Accounts for Melanopsin-Driven Responses in Mice under Diverse Lighting Conditions. PLoS One. 2013;8. doi: 10.1371/journal.pone.0053583 23301090 PMC3536742

[48] Allen AE, Storchi R, Martial FP, Bedford RA, Lucas RJ. Melanopsin Contributions to the Representation of Images in the Early Visual System. Curr Biol. 2017;27: 1623–1632.e4. doi: 10.1016/j.cub.2017.04.046 28528909 PMC5462620

[49] Shin JC, Yaguchi H, Shioiri S. Change of Color Appearance in Photopic, Mesopic and Scotopic Vision. Optical Review. 2004;11: 265–271. doi: 10.1007/s10043-004-0265-2

[50] Shin JC, Matsuki N, Yaguchi H, Shioiri S. A Color Appearance Model Applicable in Mesopic Vision. Optical Review. 2004;11: 272–278. doi: 10.1007/s10043-004-0272-3

[51] Stabio ME, Sabbah S, Quattrochi LE, Ilardi MC, Fogerson PM, Leyrer ML, et al. The M5 Cell: A Color-Opponent Intrinsically Photosensitive Retinal Ganglion Cell. Neuron. 2018;97: 150–163.e4. doi: 10.1016/j.neuron.2017.11.030 29249284 PMC5757626

[52] Lee SK, Sonoda T, Schmidt TM. M1 Intrinsically Photosensitive Retinal Ganglion Cells Integrate Rod and Melanopsin Inputs to Signal in Low Light. Cell Reports. 2019;29: 3349–3355.e2. doi: 10.1016/j.celrep.2019.11.024 31825819 PMC6951432

[53] Spitschan M, Aguirre GK, Brainard DH. Selective Stimulation of Penumbral Cones Reveals Perception in the Shadow of Retinal Blood Vessels. PLOS ONE. 2015;10: e0124328. doi: 10.1371/journal.pone.0124328 25897842 PMC4405364

[54] Zele AJ, Feigl B, Adhikari P, Maynard ML, Cao D. Melanopsin photoreception contributes to human visual detection, temporal and colour processing. Sci Rep. 2018;8: 1–10. doi: 10.1038/s41598-018-22197-w 29497109 PMC5832793

[55] Barrionuevo PA, Cao D. Contributions of rhodopsin, cone opsins, and melanopsin to postreceptoral pathways inferred from natural image statistics. J Opt Soc Am A Opt Image Sci Vis. 2014;31: A131–A139. doi: 10.1364/JOSAA.31.00A131 24695161 PMC4117214

[56] Isobe K, Motokawa K. Functional Structure of the Retinal Fovea and Maxwell’s Spot. Nature. 1955;175: 306–307. doi: 10.1038/175306a0 13235884

[57] Gardasevic M, Lucas RJ, Allen AE. Appearance of Maxwell’s spot in images rendered using a cyan primary. Vision Res. 2019;165: 72–79. doi: 10.1016/j.visres.2019.10.004 31678617 PMC6902267

[58] Tikidji-Hamburyan A, Reinhard K, Storchi R, Dietter J, Seitter H, Davis KE, et al. Rods progressively escape saturation to drive visual responses in daylight conditions. Nat Commun. 2017;8: 1813. doi: 10.1038/s41467-017-01816-6 29180667 PMC5703729

[59] Dain SJ, King-Smith PE. Thresholds for Iso-Luminous Colors Across the Visual Field. In: Drum B, Verriest G, editors. Colour Vision Deficiencies IX. Dordrecht: Springer Netherlands; 1989; p. 561–572. doi: 10.1007/978-94-009-2695-0_65

[60] Umino Y, Guo Y, Chen C-K, Pasquale R, Solessio E. Rod Photoresponse Kinetics Limit Temporal Contrast Sensitivity in Mesopic Vision. J Neurosci. 2019;39: 3041–3056. doi: 10.1523/JNEUROSCI.1404-18.2019 30737308 PMC6468098

[61] Graham DM, Wong KY. Melanopsin-expressing, Intrinsically Photosensitive Retinal Ganglion Cells (ipRGCs). In: Kolb H, Fernandez E, Nelson R, editors. Webvision: The Organization of the Retina and Visual System. Salt Lake City (UT): University of Utah Health Sciences Center; 2016. Available: http://www.ncbi.nlm.nih.gov/books/NBK27326/21413413

