# Supplementary material for: Daylights with high melanopsin stimulation appear reddish in fovea and greenish in periphery
Source: PLoS One. 2023 Apr 26;18(4):e0285053. doi: 10.1371/journal.pone.0285053 (PMC10132674; doi:10.1371/journal.pone.0285053)
Supplement: S1 Fig — (DOCX) [file pone.0285053.s001.docx]

**
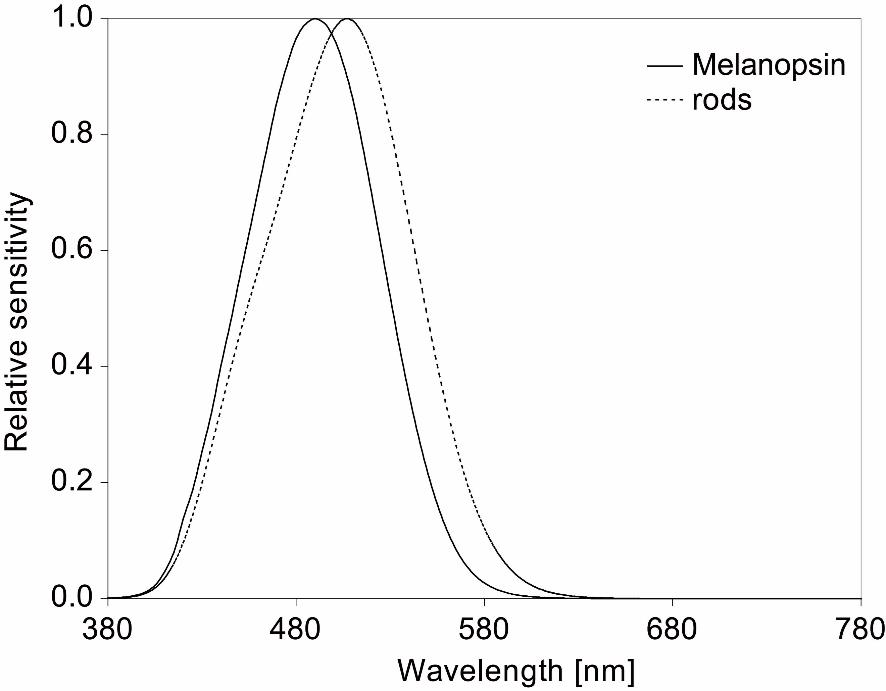
**

**S1 Fig.** Spectral sensitivities of melanopsin and rods. The spectral sensitivity functions of melanopsin and rods were referred from the International Commission on Illumination (CIE) technical report S026/E:2018 [44] and 191:2010 [43], respectively.
