# Supplementary material for: Daylights with high melanopsin stimulation appear reddish in fovea and greenish in periphery
Source: PLoS One. 2023 Apr 26;18(4):e0285053. doi: 10.1371/journal.pone.0285053 (PMC10132674; doi:10.1371/journal.pone.0285053)
Supplement: S1 Table — (DOCX) [file pone.0285053.s002.docx]

| **No.** | **Condition** | ***X*_f10_** | ***Y*_f10_** | ***Z*_f10_** | ***x*_f10_** | ***y*_f10_** |
| --- | --- | --- | --- | --- | --- | --- |
| **1** | 2700 K- | 1411  (64) | 1200  (54) | 359  (16) | 0.475  (0.478) | 0.404  (0.402) |
| **2** | 2700 K | 1380  (63) | 1224  (55) | 353  (16) | 0.467  (0.468) | 0.414  (0.413) |
| **3** | 2700 K+ | 1325  (61) | 1276  (58) | 338  (15) | 0.451  (0.452) | 0.434  (0.434) |
| **4** | 5000 K- | 1234  (56) | 1214  (55) | 946  (43) | 0.364  (0.365) | 0.358  (0.358) |
| **5** | 5000 K | 1198  (55) | 1267  (58) | 951  (43) | 0.351  (0.351) | 0.371  (0.372) |
| **6** | 5000 K+ | 1130  (52) | 1370  (63) | 944  (43) | 0.328  (0.329) | 0.398  (0.398) |
| **7** | 6500 K- | 1232  (56) | 1231  (56) | 1270  (57) | 0.330  (0.331) | 0.330  (0.330) |
| **8** | 6500 K | 1185  (54) | 1286  (59) | 1270  (58) | 0.317  (0.318) | 0.344  (0.345) |
| **9** | 6500 K+ | 1109  (51) | 1412  (64) | 1267  (57) | 0.293  (0.294) | 0.373  (0.373) |
| **10** | 8000 K- | 1230  (56) | 1229  (56) | 1501  (68) | 0.311  (0.311) | 0.310  (0.311) |
| **11** | 8000 K | 1191  (54) | 1304  (59) | 1515  (68) | 0.297  (0.298) | 0.325  (0.327) |
| **12** | 8000 K+ | 1102  (50) | 1448  (65) | 1510  (68) | 0.271  (0.273) | 0.357  (0.355) |

**S1 Table.** **Measured values of the visual stimuli calculated using the CIE 2006 10° CMFs.** The tristimulus values and chromaticity were calculated from the measured spectral power distributions using the International Commission on Illumination (CIE) 2006 10° color matching functions (CMFs) [4,5]. “No.” represents the condition number. The values without parentheses in the table denote values in higher-luminance conditions. The values in parentheses denote lower-luminance conditions. The “-,” “none,” and “+” labels correspond to half-, equal-, and two-fold the values of melanopsin/rod stimulation based on the reference illuminants of each correlated color temperature (CCT).
