# Supplementary material for: Daylights with high melanopsin stimulation appear reddish in fovea and greenish in periphery
Source: PLoS One. 2023 Apr 26;18(4):e0285053. doi: 10.1371/journal.pone.0285053 (PMC10132674; doi:10.1371/journal.pone.0285053)
Supplement: S1 File — (DOCX) [file pone.0285053.s003.docx]

**S1 Equations. Equations used in our experiment.** The tristimulus values (*X*_f_, *Y*_f,_ *Z*_f_), melanopsin/rods stimulation (*Melanopsin, rods*) and chromaticity coordinates (*x*_f_, *y*_f)_ were calculated using Eqs.1-10. The tristimulus values and the melanopsin stimulation were calculated from the measured spectral power distributions *P*(λ) in Fig 3 using the method described in the International Commission on Illumination (CIE) technical report 170-1:2006 [4], 170-2:2015 [5], S026/E:2018 [44], and 191:2010 [43]. The values are presented in Table 1.

| $L=\int\bar{l}\left( \lambda\right)P\left( \lambda\right)d\lambda$ | (1) |
| --- | --- |
| $M=\int\bar{m}\left( \lambda\right)P\left( \lambda\right)d\lambda$ | (2) |
| $S=\int\bar{s}\left( \lambda\right)P\left( \lambda\right)d\lambda$ | (3) |
| $X_{F}=683.358(1.94735469 L-1.41445123 M+0.36476327 S)$ | (4) |
| $Y_{F}=683.358(0.68990272 L+0.34832189 M)$ | (5) |
| $Z_{F}=683.358(1.93485343 S)$ | (6) |
| $x_{F}=\frac{X_{F}}{X_{F}+Y_{F}+Z_{F}}$ | (7) |
| $y_{F}=\frac{Y_{F}}{X_{F}+Y_{F}+Z_{F}}$ | (8) |
| $Melanopsin=\int S_{\mathrm{mel}}\left( \lambda\right)P\left( \lambda\right)d\lambda$ | (9) |
| $rods=\int V'\left( \lambda\right)P\left( \lambda\right)d\lambda$ | (10) |
